# Kikuchi–Fujimoto Disease Post COVID-19 Vaccination: Case Report and Review of Literature

**DOI:** 10.3390/vaccines9111251

**Published:** 2021-10-29

**Authors:** Hui Min Tan, Susan Swee-Shan Hue, Aileen Wee, Kay Choong See

**Affiliations:** 1 Department of Pathology, National University Hospital, Singapore 119074, Singapore; hui_min_tan@nuhs.edu.sg (H.M.T.); swee_shan_hue@nuhs.edu.sg (S.S.-S.H.); patweea@nus.edu.sg (A.W.); 2 Division of Respiratory and Critical Care Medicine, Department of Medicine, National University Hospital, Singapore 119074, Singapore

**Keywords:** Kikuchi–Fujimoto disease, necrotizing histiocytic lymphadenitis, COVID-19, COVID-19 vaccine, lymphadenopathy

## Abstract

With the rapid development of various coronavirus disease 2019 (COVID-19) vaccines in a bid to counter and contain the COVID-19 pandemic, unusual and uncommon side effects of COVID-19 vaccination have been increasingly reported in the literature. Ipsilateral lymphadenopathy is a fairly common side effect of vaccination of any kind, with its etiology most commonly related to reactive lymphadenopathy. However, Kikuchi–Fujimoto Disease (KFD) or necrotizing histiocytic lymphadenitis is rarely observed post-vaccination, with only one other case of KFD post COVID-19 vaccination reported to date. We report two more cases of KFD post COVID-19 vaccination in the Asian population, highlighting the clinical course and salient clinical, radiological and histologic findings. In addition, we provide a literature review of the existing cases of lymphadenopathy post COVID-19 vaccination with cytologic and/or histologic correlation.

## 1. Introduction

More than a year after the coronavirus disease 2019 (COVID-19) pandemic emerged and rocked the world, many COVID-19 vaccines, ranging from messenger RNA (mRNA)-based vaccines to inactivated virus vaccines, have been developed at unprecedented speed. As various countries ramp up their vaccination rates in a bid to contain the pandemic, unusual and uncommon side effects of COVID-19 vaccines have been increasingly discovered and reported.

Ipsilateral lymphadenopathy following vaccination is a well-described side effect and has been reported with several types of vaccines [1]. As part of the immune system, lymph nodes located at the drainage basin of the site of vaccination may undergo reactive lymphoid hyperplasia following stimulation by the antigens within the vaccine. In particular, post COVID-19 vaccination with mRNA-based vaccines, close to 15% of patients reported unilateral axillary lymphadenopathy [2]. For case reports that included some form of cytologic or histologic confirmation, most observed that the cause of lymphadenopathy was reactive in nature. In contrast, Kikuchi–Fujimoto Disease (KFD) or necrotizing histiocytic lymphadenitis is a very rare occurrence with only one published case report to date [3]. We describe two more patients from our institution who developed histologically confirmed KFD after receiving mRNA-based COVID-19 vaccine (Pfizer-BioNTech).

## 2. Case Report

**Figure 1:**
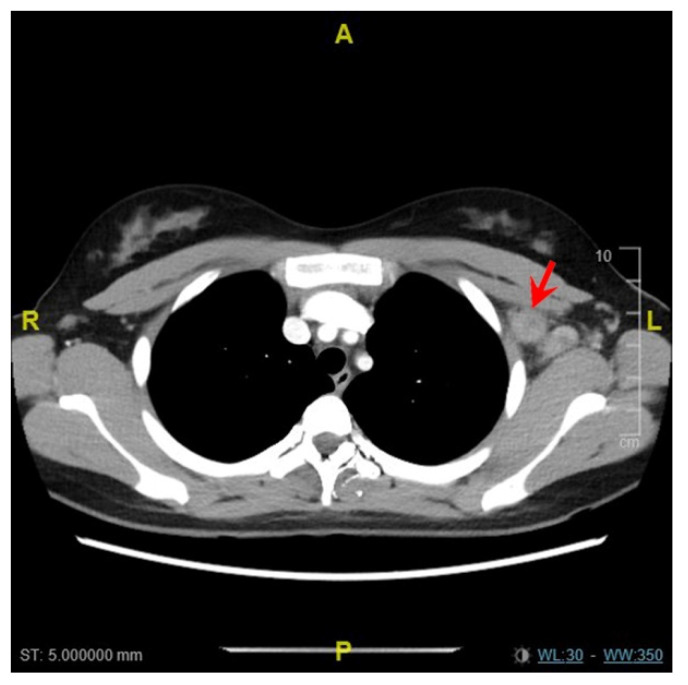
Computed tomography scan of patient A demonstrating enlarged left subpectoral and axillary lymph nodes (red arrow).

**Figure 2:**
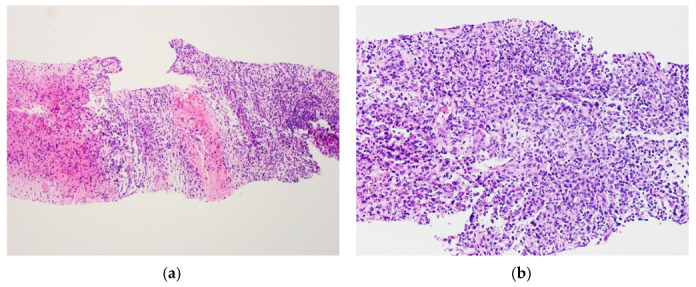
Histologic findings from core biopsy of left axillary lymph node from patient A. Formalin-fixed, paraffin-embedded (FFPE) sections of the lymph node biopsy were stained with hematoxylin and eosin (H&E). The lymph node showed confluent necrosis (**a**, left aspect) surrounded by a polymorphous population of lymphoid cells admixed with karyorrhectic debris and crescentic histiocytes (**b**).

The first patient (patient A) was an 18 year old Asian woman who was previously well and received her first vaccination dose on 4 June 2021 (Day 1). She developed fever and left axillary lymphadenopathy on Day 35, which persisted until presentation on Day 42. The maximum temperature recorded was 40.5 degrees Celsius on Day 49. Transient leukopenia developed with a nadir on Day 52 (leukocyte count 3.26 × 10^9^/L, neutrophil count 1.37 × 10^9^/L). SARS-CoV-2 nucleocapsid antibody was not detected, and antibody titer against spike protein was 133.0 U/mL. Anti-nuclear antibody titer was <1:80, and anti-ds-DNA titer was 10 IU/mL (normal <100 IU/mL). A computed tomography (CT) scan of the neck and thorax performed on Day 49 demonstrated enlarged left supraclavicular, subpectoral and axillary lymph nodes measuring 1.7 to 2.0 cm (Figure 1). Ultrasound-guided left axillary lymph node core biopsy performed on Day 53 provided a diagnosis of necrotizing histiocytic lymphadenitis that was consistent with KFD (Figure 2). Ziehl-Neelsen (ZN) and Gomori methenamine silver (GMS) stains had been performed on the histology specimen, and no acid-fast bacilli or fungal organisms were identified, respectively.

**Figure 3:**
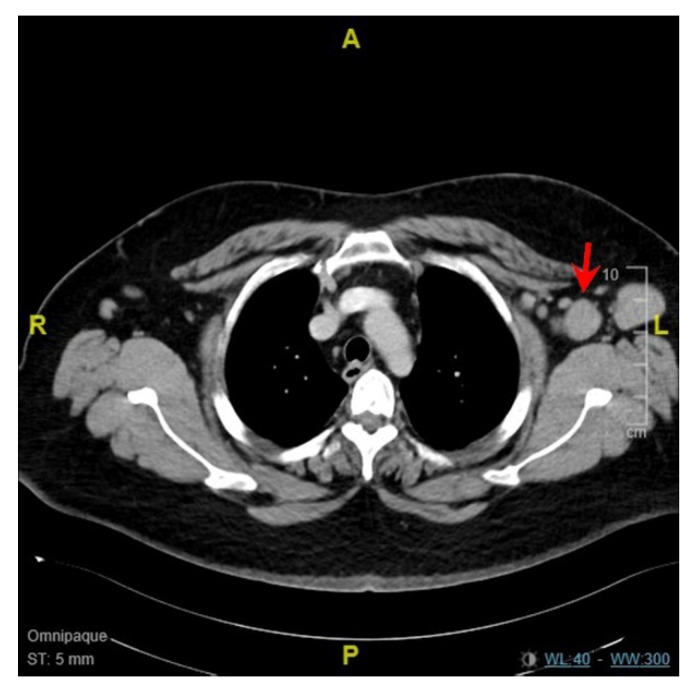
Computed tomography scan of patient B demonstrating enlarged left axillary lymph nodes (red arrow).

**Figure 4:**
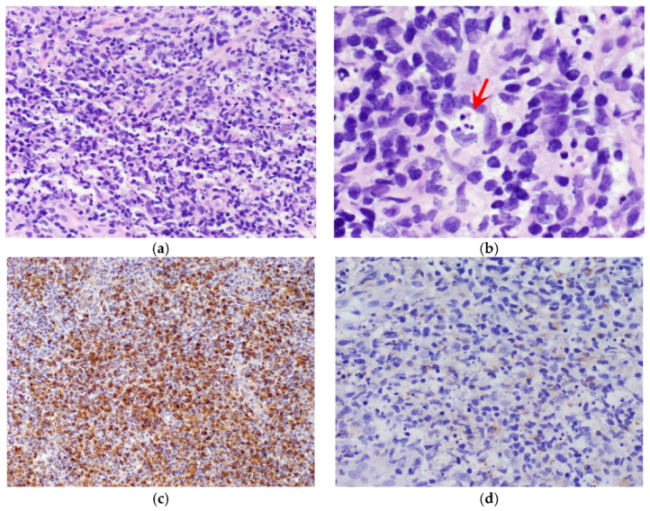
Histologic findings from core biopsy of left axillary lymph node from patient B. FFPE sections of the lymph node biopsy were stained with H&E. (**a**) The lymph node showed numerous histiocytes, including some with crescentic morphology (**b**, red arrow), admixed with karyorrhectic debris, plasmacytoid monocytes and small lymphocytes. Immuno-peroxidase staining for CD68 (**c**) highlighted the histiocytes, which also showed focal expression of myeloperoxidase (MPO) (**d**).

The second patient (patient B) was a 34 year old Asian man with diabetes mellitus and hypertension who received his first vaccination dose on 3 July 2021 (Day 1). He developed fever and left axillary lymphadenopathy on Day 17, persisting until presentation on Day 23. The maximum temperature recorded was 38.9 degrees Celsius on Day 26. Transient leukopenia developed with a nadir on Day 39 (leukocyte count 1.79 × 10^9^/L, neutrophil count 1.05 × 10^9^/L). SARS-CoV-2 nucleocapsid antibody was not detected, and antibody titer against spike protein was 6.9 U/mL. Anti-nuclear antibody titer was 1:80, and anti-ds-DNA titer was <3 IU/mL. A CT scan of the thorax, abdomen and pelvis performed on Day 27 demonstrated enlarged left axillary lymph nodes measuring up to 3.2 cm (Figure 3). Ultrasound-guided left axillary lymph node core biopsy performed on Day 33 provided a diagnosis of histiocytic lymphadenitis that was consistent with KFD (Figure 4). ZN and GMS stains had been performed on the histology specimen, and no acid-fast bacilli or fungal organisms were identified, respectively.

In both cases, COVID-19 ribonucleic acid (RNA) polymerase chain reaction (PCR), viral testing (human immunodeficiency virus antibody, Epstein–Barr virus IgM, cytomegalovirus IgM, toxoplasma IgM, dengue IgM, hepatitis B surface antigen and anti-hepatitis C antibody) and blood cultures were negative. Peripheral blood film did not show any blasts or atypical lymphocytes. The clinical course was marked by high fever and significant ipsilateral lymphadenopathy developing 2–5 weeks after vaccination and transient leukopenia developing 4–7 weeks after vaccination. The fever did not respond to paracetamol. Rather, a non-steroidal anti-inflammatory agent (ibuprofen) resulted in rapid and complete resolution of fever and lymphadenopathy over several days, with patient A’s symptoms resolving by Day 58 and patient B’s symptoms resolving by Day 38.

## 3. Discussion

KFD is an uncommon but benign cause of lymphadenopathy. First described in the 1970s, it is more commonly observed in the Asian population and usually affects young adults. Patients typically present tender lymphadenopathy affecting the cervical (most common), axillary and/or supraclavicular lymph nodes, as well as fever. Other systemic symptoms may also be present, rendering KFD a great mimicker of various infectious, autoimmune or even neoplastic conditions. While KFD has a self-limiting course and treatment is mainly symptomatic in nature, the exact etiology remains uncertain with postulations mainly related to some form of exaggerated T-cell mediated immune response to antigens [4,5,6].

KFD very uncommonly occurs following vaccination, with only rare reports in the literature describing its occurrence after influenza vaccination [7], human papillomavirus vaccination and Japanese encephalitis virus vaccination [8]. A literature search yielded only one case report of KFD following COVID-19 vaccination at the time of writing [3], with many other papers describing cytologic or histologic findings of reactive follicular hyperplasia or reactive lymphadenopathy [9,10,11,12,13,14]. Table 1 details the key features of published cases of lymphadenopathy post COVID-19 vaccination, which also had cytologic and/or histologic follow-up.

In the patients who developed reactive lymphadenopathy post COVID-19 vaccination, they usually had no symptoms or had only experienced painless enlargement of lymph node(s). On the contrary, the patients with KFD post COVID-19 vaccination had fever and other systemic manifestations. In addition, reactive lymphadenopathy post COVID-19 vaccination may occur as soon as the day after the vaccine was given, but KFD tended to occur in a more delayed setting, ranging from 10 to 35 days based on our two cases and the sole other published case.

Certain infectious lymphadenitis, autoimmune conditions (e.g., systemic lupus erythematosus in particular) and lymphoproliferative disorders may also give rise to similar or overlapping histomorphologic findings as KFD, and these have to be considered and excluded before a diagnosis of KFD is made. In our two cases, extensive clinical workup was performed to exclude infectious and autoimmune causes, and histology also confirmed the absence of lymphoproliferative disorders.

Being a self-limiting disease, KFD typically resolves within months. While there is no specific treatment for KFD, symptomatic treatment with analgesics is usually sufficient, with steroids sometimes being used in more severe or recurrent cases [5,6]. In our two cases as well as the other case reported in literature by Soub et al. [3], the patients’ symptoms resolved within a month of onset, and treatment with analgesics proved to be effective for the relief of symptoms. For our two cases, non-steroidal anti-inflammatory drugs (NSAIDs), in particular, appeared to be more effective than paracetamol. Epidemiology-wise, our cases included young Asian adults, while the case reported by Soub et al. was a young Middle Eastern male.

We note that the patients who developed KFD post COVID-19 vaccination all received mRNA-based vaccines. Nonetheless, more data are needed to determine if KFD is an occurrence specific to mRNA-based vaccines only or if KFD could also occur in patients receiving other types of vaccines.

In addition, KFD can occur post or concurrent with COVID-19 infection [15,16]. While the ethnicity of the patients was not specifically mentioned in the available case reports, the patients were young (less than 20 years old), presented with prolonged fever and other systemic symptoms and one case mentioned rapid resolution of the symptoms within 7 days of commencing supportive treatment [15,16]. The clinical course of these cases was similar to that of KFD in general, as well as the cases of KFD post COVID-19 vaccination.

While the limited number of cases available renders it difficult to determine if the association of KFD and COVID-19 infection/vaccination is that of a causal relationship, KFD remains an important condition to consider when patients with recent COVID-19 vaccination or infection develop lymphadenopathy with other systemic symptoms.

## 4. Conclusions

KFD may rarely occur after COVID-19 vaccination, with patients usually presenting with lymphadenopathy, fever and other systemic symptoms more than a week after the vaccine dose was applied. The initial clinical presentation may be alarming and mimics infections, autoimmune conditions or even lymphoproliferative disorders, especially if the history of prior vaccination was not readily provided or considered. Fortunately, the disease runs a benign and self-limiting course with rapid resolution of symptoms after symptomatic and supportive treatment. It is, hence, important that clinicians recognize this potential condition that may occur after COVID-19 vaccination and to avoid excessive investigation and unnecessary treatment.

## Figures and Tables

**Table 1:** Key features of cases of lymphadenopathy post COVID-19 vaccination with cytologic and/or histologic follow-up.

Author	Age	Gender	Vaccine Received	Interval between Vaccination and LAD ^1^ or First Symptom	Site(s) of LAD *	Type of Sampling ^†^	Diagnosis
Our case, patient A	18	Female	Pfizer- BioNTech	35 days	Supraclavicular, subpectoral and axillary	Core biopsy	KFD ^2^
Our case, patient B	34	Male	Pfizer- BioNTech	17 days	Axillary	Core biopsy	KFD
Soub et al. (August 2021) [3]	18	Male	Pfizer- BioNTech	10 days	Supraclavicular, cervical and axillary	Excision biopsy	KFD
Özütemiz et al. (February 2021) [9]; oncologic patients	32	Female	Pfizer- BioNTech	6 days	Axillary	Nil	–
	57	Female	Pfizer- BioNTech	5 days	Axillary	Nil	–
	41	Male	Pfizer- BioNTech	4 days	Axillary	Nil	–
	46	Female	Pfizer- BioNTech	15 days	Axillary, supraclavicular	Nil	Reactive lymph node
	38	Female	Pfizer- BioNTech	8 days	Axillary	Core biopsy	Reactive follicular hyperplasia
Cardoso et al. (May 2021) [10]	48	Female	Pfizer- BioNTech	2 weeks	Cervical	Excision biopsy	Reactive follicular hyperplasia
Placke et al. (June 2021) [11]; patients with previous skin cancer	54	Female	CureVac	30 days	Axillary	Excision biopsy (sentinel node)	Lymphofollicular hyperplasia
	28	Female	Pfizer- BioNTech	28 days	Axillary	Excision biopsy (selective)	Sarcoid-like reaction
	58	Male	Pfizer- BioNTech	7 days	Axillary	Excision biopsy (selective)	Not mentioned, but non-malignant
	77	Male	Pfizer- BioNTech	11 days	Axillary	Excision biopsy (sentinel node)	Not mentioned, but non-malignant
	91	Male	Pfizer- BioNTech	16 days	Axillary	Excision biopsy (sentinel node)	Not mentioned, but non-malignant
	44	Male	Pfizer- BioNTech	15 days	Axillary	Excision biopsy (sentinel node)	Not mentioned, but non-malignant
	43	Female	Pfizer- BioNTech	50 days	Axillary	Lymphadenectomy	Not mentioned, but non-malignant
	84	Female	Pfizer- BioNTech	12 days	Axillary	Excision biopsy (sentinel node)	Not mentioned, but non-malignant
Hagen et al. (July 2021) [12]	66	Male	Moderna	22 days	Axillary	FNAC ^3^	Reactive lymphadenopathy
	41	Female	Moderna	3 days	Infraclavicular	FNAC	Reactive lymphadenopathy
	47	Female	Pfizer- BioNTech	19 days	Supraclavicular	FNAC	Reactive lymphadenopathy
	47	Female	Moderna	8 days	Cervical	FNAC	Reactive lymphadenopathy
	52	Male	Pfizer- BioNTech	12 days	Retroclavicular (contralateral)	FNAC	Negative for malignancy
Tintle and Chen (July 2021) [13]	23	Female	Moderna	1 week	Axillary, intra-abdominal	Excision biopsy	Lymphoid and Langerhan cell hyperplasia, hemophagocytosis
Tan et al. (August 2021) [14]	34	Male	Pfizer- BioNTech	1 day	Supraclavicular	FNAC	Reactive lymphadenopathy

^1^ LAD = lymphadenopathy. ^2^ KFD = Kikuchi–Fujimoto disease. ^3^ FNAC = fine needle aspiration cytology. * Site(s) of LAD are all presumed to be ipsilateral unless otherwise specified. ^†^ For cases with initial FNAC followed by tissue biopsy, only the latter is indicated.

## Data Availability

Data sharing not applicable.
